# Influence of Long-Term Storage on Shape Memory Performance and Mechanical Behavior of Pre-stretched Commercial Poly(methyl methacrylate) (PMMA)

**DOI:** 10.3390/polym11121978

**Published:** 2019-12-01

**Authors:** Changchun Wang, Yuming Dai, Bo Kou, Wei Min Huang

**Affiliations:** 1 Jiangsu key laboratory of advanced structural materials & application technology, School of Material Science and Engineering, Nanjing Institute of Technology, Nanjing 211167, China; ccwang@njit.edu.cn (C.W.); Y.M.Dai@hotmail.com (Y.D.); koutianbo@hotmail.com (B.K.); 2 School of Mechanical and Aerospace Engineering, Nanyang Technological University, 50 Nanyang Avenue, Singapore 639798, Singapore

**Keywords:** shape memory polymer, storage, shape memory performance, mechanical behavior, poly(methyl methacrylate), shape fixity ratio, shape recovery ratio

## Abstract

In this paper, we experimentally investigate the influence of storage at 40 °C on the shape memory performance and mechanical behavior of a pre-stretched commercial poly(methyl methacrylate) (PMMA). This is to simulate the scenario in many applications. Although this is a very important topic in engineering practice, it has rarely been touched upon so far. The shape memory performance is characterized in terms of the shape fixity ratio (after up to one year of storage) and shape recovery ratio (upon heating to previous programming temperature). Programming in the mode of uniaxial tension is carried out at a temperature within the glass transition range to one of four prescribed programming strains (namely 10%, 20%, 40% and 80%). Also investigated is the residual strain after heating for shape recovery. The characterization of the mechanical behavior of programmed samples after storage for up to three months is via cyclic uniaxial tensile test. It is concluded that from an engineering application point view, for this particular PMMA, programming should be done at higher temperatures (i.e., above its *T_g_* of 110 °C) in order to not only achieve reliable and better shape memory performance, but also minimize the influence of storage on the shape memory performance and mechanical behavior of the programmed material. This finding provides a useful guide for engineering applications of shape memory polymers, in particular based on the multiple-shape memory effect, temperature memory effect, and/or low temperature programming.

## 1. Introduction

The shape memory effect (SME) refers to the ability of a material to return its original shape, but only at the presence of a right stimulus, such as heat, chemical or light, etc. [1,2,3,4,5]. In addition to a lot of purposely developed ones [6,7,8,9,10,11], many conventional engineering polymers have been confirmed to have excellent SME, in particular heat-induced (heating-responsive) SME [12,13,14,15,16,17,18,19,20]. The phenomenon of the SME provides an additional dimension to reshape product design in many ways [6,21,22]. We have seen more and more applications of shape memory polymers (SMPs) in recent years [23,24,25,26,27,28,29,30]. 

A full shape memory cycle includes two steps, namely programming, which is to fix the temporary shape, and recovery, which is to apply the stimulus to active the SME. From a real engineering application point of view, such as in active disassembly [31,32], deployable structures [33] and anti-counterfeit applications [34,35,36,37,38,39,40], activation of the SME may not be carried out right after programming, but after a period of storage. As such, we need to consider the influence of aging at around room temperature after programming [41], which is a topic that has been less explored so far [42,43], but that is utterly important from an engineering application point of view.

The purpose of this paper is to experimentally investigate the influence of storage at 40 °C on the shape memory performance and mechanical behavior of a commercial poly(methyl methacrylate) (PMMA), which is a typical engineering polymer, used in many applications, including optical lens [44,45] and plastic screw. This particular PMMA has been verified to have excellent heating-responsive SME in our previous studies (e.g., in [46,47]), and unlike some moisture/water-responsive SMPs (e.g., the polyurethane reported in [48]), it appears to be non-sensitive to moisture in air. PMMA and polycarbonates (PC) are typical amorphous polymer, and their SME was reported over 20 years ago [49,50]. Both experimental investigation and simulation on the SME of amorphous polymers (without chemical cross-linking, but via physical cross-linking) have been well documented in the literature (e.g., in [41,51,52,53]). Different programming temperatures (all within the glass transition range) and programming strains (up to 80%, in the mode of uniaxial tension) are applied in this study, as they have been identified to be influential parameters on the shape memory performance of SMPs [17]. Since according to our previous studies, this commercial PMMA is able to almost fully recover its original shape upon heating to over its glass transition temperature range, shape recovery is activated by heating to the previous programming temperature in order to explore the so-called temperature memory effect (TME) [12,54,55], which is potentially applicable in, for instance, step-by-step active disassembly [46]. The shape memory performance of programmed samples (after up to one year of storage) is characterized in terms of the shape fixity ratio (right after programming or after storage), shape recovery ratio and residual strain after shape recovery. Mechanical behavior of the programmed samples right after programming or after a period of storage (up to three months) is characterized via cyclic uniaxial tensile test at room temperature. Comparison between the experimental results plotted in different ways (only one presented here and the rest are presented in the Supplementary file) reveals some general trends, which provide a useful guide in real engineering applications of SMPs.

## 2. Material, Sample Preparation and Experimental

### 2.1. Material and Sample Preparation

**Figure 1:**
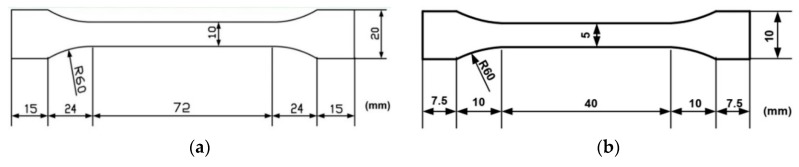
Dog-bone shaped samples for characterization of (**a**) shape memory performance and (**b**) mechanical behavior (via cyclic uniaxial tension).

PMMA sheet of 1 mm thickness from Ying Kwang Acrylic, Singapore (same type as used in [46,47]) was laser cut into dog-bone shaped samples with two different dimensions, one for characterization of the shape memory performance and the other for cyclic uniaxial tension to characterize the mechanical behavior right after programming or after storage (refer to Figure 1). A small piece (about 10 mg) was also prepared for a differential scanning calorimeter (DSC) test.

### 2.2. Differential Scanning Calorimetry (DSC)

**Figure 2:**
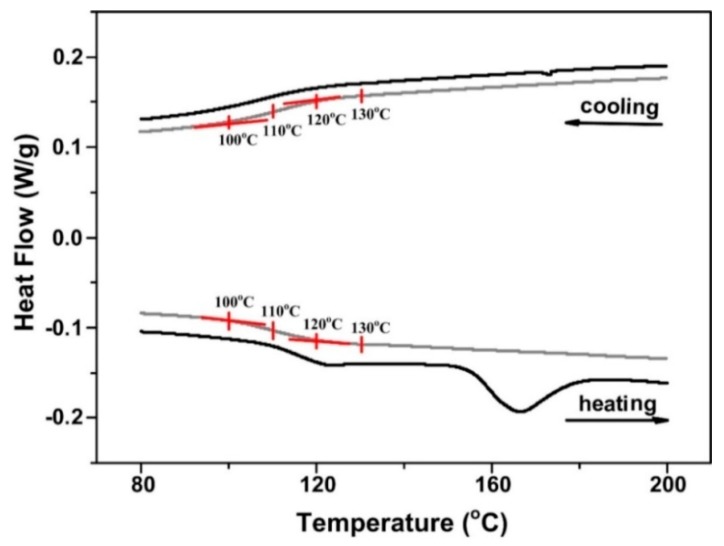
Differential scanning calorimetry (DSC) result of two continuous thermal cycles. Black line: 1st cycle; grey line: 2nd cycle. Only the result within 80 to 200 °C is presented.

DSC test was conducted at a heating/cooling rate of 5 °C min^−1^ between 20 and 210 °C for two cycles using DSC, TA Q200 instrument, USA. As revealed in Figure 2 (only the result within 80 °C to 200 °C is presented for a better view), in the 1st heating process, there are two transitions, while in the subsequent cooling process, there is only one transition at about the same temperature range as that for the 1st transition in the 1st heating process (between 100 and 130 °C), which is identified as the glass transition. In the 2nd heating process, the 2nd transition observed in the 1st heating process disappears, while the rest of the curve is more or less the same as that in the 1st thermal cycle. After over two months of room temperature storage, it is observed that this 2nd transition reappears (refer to Figure S1 in Supplementary Materials). Since PMMA is a typical amorphous polymer, the 2nd transition observed in the 1st heating process should be related to the fabrication process (e.g., additional chemicals for easy extrusion). According to the DSC result of the 2nd thermal cycle, the glass transition temperature (*T*_g_) of this PMMA may be determined as 110 °C. If thermal cycling is below 150 °C, there is no change in the heat flow versus temperature relationship in both the heating process and the cooling process (refer to Figure S2 in Supplementary Materials). Hence, the maximum temperature in the course of this study to investigate the SME was 130 °C.

### 2.3. Shape Memory Performance

All samples (refer to Figure 1a for dimensions) were annealed at 120 °C for two hours to eliminate the influence of previous thermal history. Programming (in the mode of uniaxial tension) was conducted using an Instron 5565 at a strain rate of 0.2%/s. Four temperatures (100, 110, 120 and 130 °C, all within the glass transition range) were selected as the programming temperature and four programming strains (10%, 20%, 40% and 80%) were used. After cooling and removal of the clamps, the samples were kept inside an oven at 40 °C, which was slightly above the ambient temperature (about 22 °C), in order to simulate the upper-end possible scenario in many engineering applications. After a predetermined period of storage time (up to one year), the samples were taken out and then heated to their previous programming temperature for shape recovery. We did not heat the programmed samples in a step-by-step manner as in our previous works to characterize other engineering polymers [13,14,15,16], since the shape recovery of this PMMA upon heating to 130 °C is always about 100% [46,47,56]. Each test was repeated four times. 

Note that the stress and strain mentioned herein are meant for engineering stress and engineering strain. Thermal strain is relatively small and is ignored for simplicity.

In a typical shape memory cycle, assume that the original length (e.g., the gauge length, which is the middle part of dog-bone shaped sample as shown in Figure 1) is *l*_0_. After heating to the required programming temperature, the sample is extended to *l*_1_. The length of the sample becomes *l*_2_ after it is cooled back to room temperature and unloaded. The length of the sample changes to *l*_3_ upon reheating to the previous programming temperature. The residual strain can be calculated by,

ε*_s_*= (*l*_3_ − *l*_0_)/*l*_0._ (1)

In many real engineering applications, the shape/dimension change is of more to us. As such, instead of following the conventional definitions based on strain as in, for instance [57], we use length to characterize the shape memory performance. Hence, the shape fixity ratio (*R*_f_) is defined by,

*R*_f_ = *l*_2_/*l*_1_ (2)

Since in this study, after the above-mentioned cooling back to room temperature and then unloading, the samples were stored at 40 °C for a required period of time to simulate the real scenario in many engineering applications, the free-standing material may go through relaxation over the period of storage. Therefore, *l*_2_ used in the current study is the value measured right before heating for shape recovery. As for the shape recovery ratio (*R_r_*), it is defined by,

*R*_r_ = (*l*_2_ − *l*_3_)/(*l*_2_ − *l*_0_). (3)

### 2.4. Mechanical Characterization

Mechanical characterization of the samples after programming and storage was via cyclic uniaxial tensile test at room temperature. Following the same procedure mentioned in Section 2.3, samples (refer to Figure 1b for dimensions) were prepared and then also kept in the oven at 40 °C for a required period of time (up to three months) before going through a cyclic uniaxial tensile test at room temperature. A constant strain rate of 0.2%/s was applied in both loading and unloading. In all cyclic tests, the samples were stretched by 1% in the first cycle and then to 3% strain in the second cycle. Subsequently, the samples were further stretched to fracture. One experiment was carried out for one set of parameters (i.e., programming strain, programming temperature and storage time).

## 3. Results and Discussions

### 3.1. Shape Memory Performance

Besides programming temperature and programming strain, which have been identified to have strong influence on the shape memory performance of polymeric materials [17], storage time is an additional parameter in the current study.

**Figure 3:**
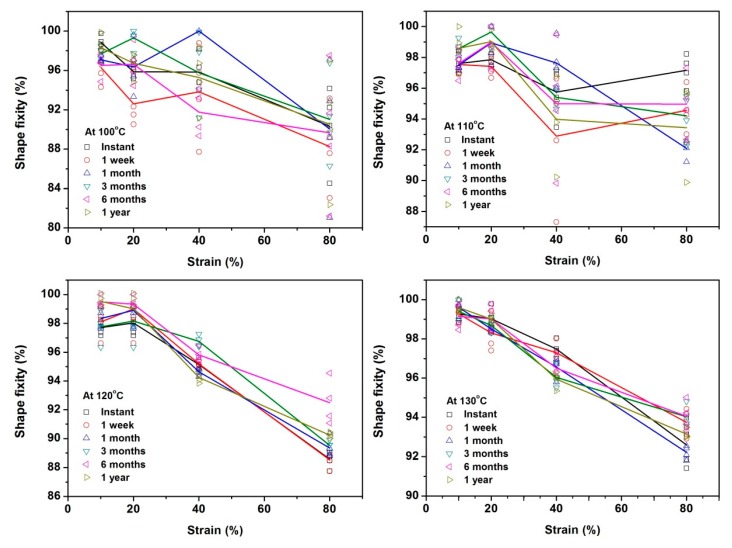
Shape fixity ratio (%) against programming strain. Symbols are used for the individual experimental result, and solid lines of the same color as the symbols indicate the average.

In order to clearly reveal the actual influence of all these three parameters, the shape fixity ratios of all tested samples are plotted in three ways, against the programming temperature (Figure S3 in Supplementary Materials), programming strain (Figure 3) and storage time (Figure S4 in Supplementary Materials), respectively. As mentioned above, each type of test was repeated four times. In order to reveal the actual fluctuation, we plot all experimental results together with the average. Herein, symbols are used for the individual experimental result, and solid lines of the same color as the symbols indicate the average. Since the material used here is a commercial engineering polymer, the shape memory performance, which is of our interest in this study, is not within the concerns for quality control in current commercial manufacturing process. Hence, we will focus more on the average to reveal the general trend.

Despite some occasional relatively significant fluctuation, a couple of general trends can be observed. 

We can see from Figure S3, with the increase in programming strain, shape fixity ratio drops, from over 96% (10% programming strain) down to over 88% (80% programming strain). As for the influnce of programming temperature, on the one hand, programming at higher temperatures effectively minimizes the influnece of storage time on the shape fixity ratio, in particular, if the applied programming strain is small. Figure 3 confirms that the shape fixity ratio decreases with the increase in programming strain. Figure S4 clearly reveals the influence of storage time, i.e., while occasionally the shape fixity ratio may fluctuate up to 8%, in general, storage may cause the shape fixity ratio to vary by 2% or less.

**Figure 4:**
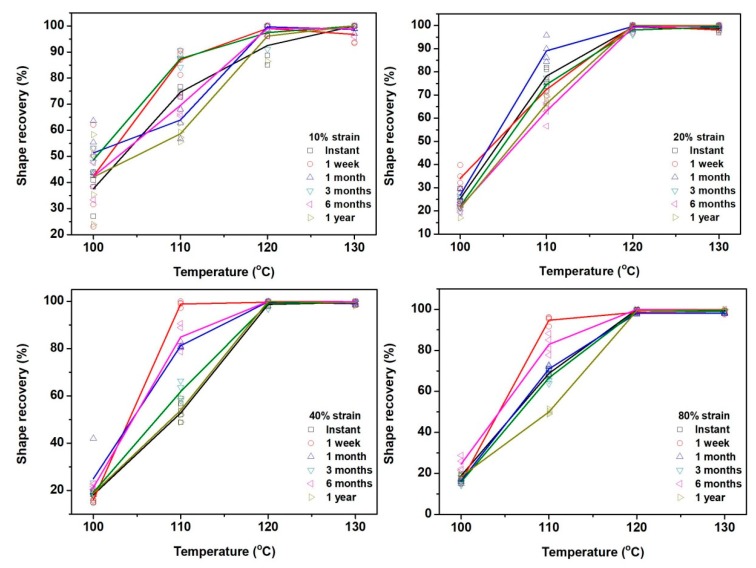
Shape recovery ratio (%) against programming temperature. Symbols are used for individual experimental result, and solid lines of the same color as the symbols indicate the average.

Figure 4 presents the exerimental results of the shape recovery ratio, which were obtained upon heating to their respective programming temperature. It plots the relationships between the shape recovery ratio (%) and programming temperature upon stretching to different programming strains, which confirms the influence of programming strain and programming temperature on shape recovery previously reported in [17], i.e.,

(a) The increase in programming strain may result in less shape recovery upon heating to the previous programming temperature.
(b) The increase in programming temperature may result in better shape recovery upon heating to the previous programming temperature.

The influnce of storage time is minimized if programming is carried out at higher temperatures. If programming temperature is 120 °C or above, the corresponding shape recovery is always about 100%. Slightly less than 100% shape recovery in samples with a programming strain of 10% and programming temperatures of 120 or 130 °C could be due to minor inaccuracy in measuring as the programmig strain of 10% is small. 

In Figure S5 (in Supplementary Materials), we replot the shape recovery ratio (%) as a function of storage time, which, in addition to confirm that with the increase in programming temperature, shape recovery increases, the followings are observed,

(a) If the programming temperature is 120 °C and above, shape recovery ratio is close to 100% for most programming strains and storage time.
(b) Programming at 100 °C results in much less shape recovery, mostly less than 50%. While the influence of storage may cause fluctuation in shape recovery ratio by over 10% when the applied programming strain is small, the increase in programming strain tends to decrease the shape recovery ratio.
(c) Programming at 110 °C results in remarkable fluctuation in the shape recovery ratio, although in general, an extended storage time slightly decreases the shape recovery ratio.

**Figure 5:**
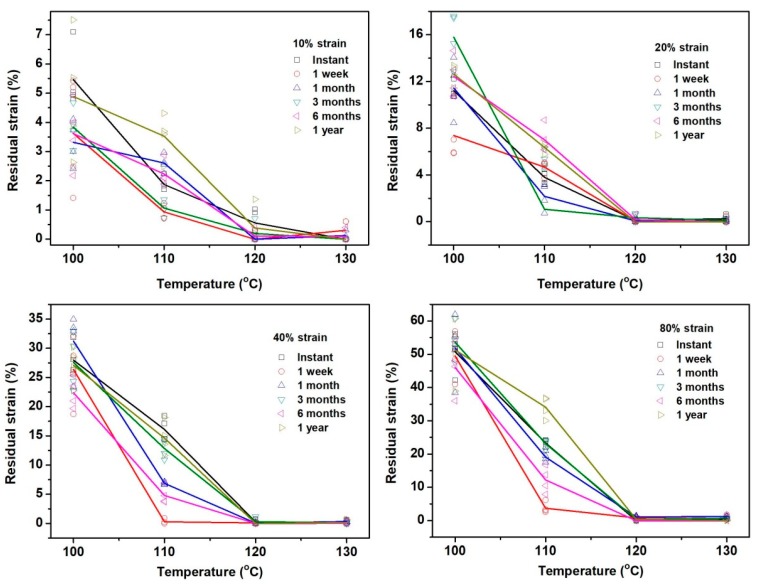
Residual strain against programming temperature. Symbols are used for individual experimental result, and solid lines of the same color as the symbols indicate the average.

Residual strain at the end of a full shape memory cycle is plotted against programming temperature in Figure 5 or against storage time in Figure S6 (in Supplementary Materials). Figure 5 reveals clearly that with the increase in programming strain, the residual strain in the samples programmed at lower temperatures increases, while that in the samples programmed at higher temperatures is mostly very close to zero, i.e., about full shape recovery. The influence of storage time is mostly remarkable only in the samples programmed at lower temperatures. Again, it appears that programming at 110 °C results in not only remarkable residual strain (but less than that programmed at 100 °C), but also even more significant fluctuation than that programmed at 100 °C after storage. When we plot the residual strain as a function of storage time in Figure S6, it confirms that high-temperature programming (at 120 and 130 °C) is able to effectively improve shape recovery. If programming is carried out at 100 °C, the residual strain depends on the programming strain. A higher programming strain results in a higher residual strain, but the influence of storage time seemingly only causes fluctuation by around 5% strain in the residual strain. Remarkable fluctuation is observed in samples programming at 110 °C, although the actual value of residual strain is less than that programmed at 100 °C. It should be pointed out that it appears to be very hard to predict the relationship between residual strain and storage time in samples stretched to larger programming strains at 110 °C.

At this point, we may conclude that from engineering application point view, for this particular PMMA, programming should be done at higher temperatures (i.e., above its *T*_g_ of 110 °C) in order to achieve reliable and better shape memory performance. Consequently, the influence of storage time can be largely ignored.

### 3.2. Mechanical Behavior 

**Figure 6:**
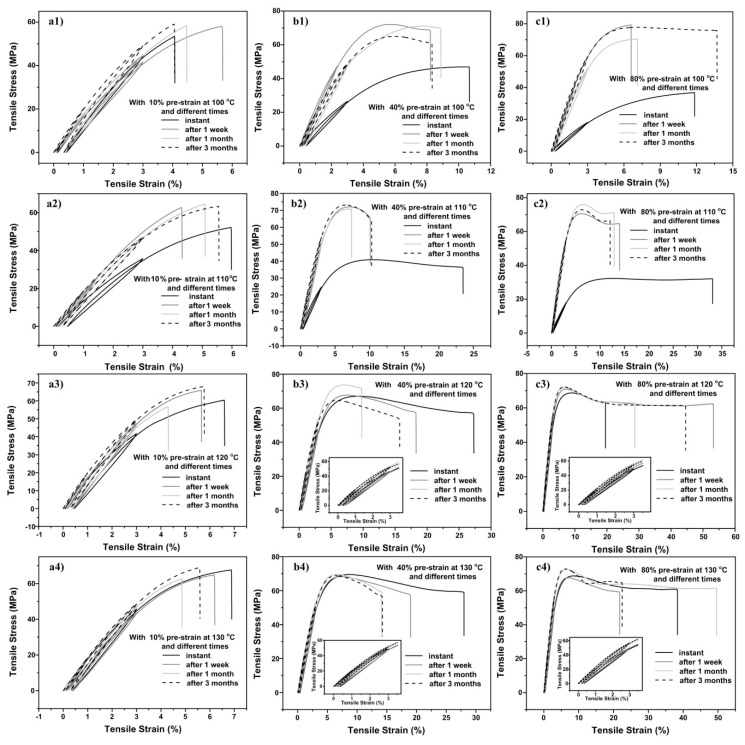
Stress versus strain relationship in cyclic uniaxial tension. (Inset of zoom-in view of the small strain range is included if necessary).

The stress versus strain relationships of all samples after programming and storage are presented in Figure S7 (in Supplementary Materials) and Figure 6. For convenience, same as in Section 3.1, we plot the results in two different ways for easy comparison of the influence of programming strain (Figure S7I,II) and the influence of storage time (Figure 6), respectively. Herein, the stress and strain are calculated using the updated cross-sectional area and the gauge length of individual programmed sample before each cyclic test.

According to Figure S7I,II in which the stress versus strain curves of all samples programmed at the same temperature but to different strains are plotted together, it appears that with the increase in programming strain, the material tends to become less brittle, and the increase in programming temperature also helps to improve the ductility of the material. A closer-look reveals that if programming is carried out at lower temperatures, with the increase in programming strain, as-programmed material becomes softer in the early loading stage, which is opposite to what is observed in the samples programmed at high temperatures. Those softened pieces gradually harden during storage, and become even harder than the less stretched ones. Programming via stretching at higher temperatures to larger strains actually improves the ductility of the as-programmed material, while the corresponding hardness (i.e., the Young′s modulus) does not change much, perhaps slightly increasing.

In Figure 6, the stress versus strain curves of all samples programmed at the same temperature to the same programming strain are plotted together to show the effects of storage. This confirms that in general, programming at higher temperatures (above *T*_g_) results in a rather stable stress versus strain relationship in the early stage before yielding even after three months of storage. As-programmed material becomes significantly softer and more ductile right after programming at lower temperatures (*T*_g_ and below). Storage at 40 °C over a week hardens the material remarkably.

At this point, it appears that programming at lower temperatures softens the material and the material also turns to be ductile. With the increase in programming strain, such phenomena become more remarkable. On the other hand, programming at higher temperatures only tends to increase the ductility of the material. Storage does alter the mechanical properties of the programmed samples, i.e., the material becomes harder and less ductile for samples programmed at lower temperatures; while samples programmed at higher temperatures become less ductile only (as they do not apparently soften after programming). It takes about one week to stabilize the material programmed at lower temperatures, while after three months, the material programmed at higher temperatures is seemingly still in the process of stabilization. Thus, it appears that programming at higher temperatures is able to effectively minimize the influence of storage time on the stress versus strain curve (before yielding) of the programmed material.

## 4. Conclusions

We experimentally investigate the influence of storage at 40 °C on a commercial PMMA after programming via uniaxial tension at different temperatures (within the glass transition range) and to different strains (up to 80%). The shape memory performance (in terms of the shape fixity ratio, shape recovery ratio and residual strain) of samples after up to one year of storage is revealed. The influence of storage (up to three month) on the mechanical behavior of programmed samples via cyclic uniaxial tensile is obtained. It is concluded that for this particular PMMA, programming should be done at higher temperatures (i.e., above its *T*_g_ of 110 °C) in order to achieve reliable and better shape memory performance and negligible influence of storage time on the shape memory performance and mechanical behavior (in term of the stress versus strain curve before yielding) in programmed material. This finding provides useful guide for engineering applications of SMPs, in particular based on the multiple-shape memory effect, temperature memory effect and/or low temperature programming. 

## Supplementary Materials

The following are available online at https://www.mdpi.com/2073-4360/11/12/1978/s1, Figure S1: DSC result of pre-heated PMMA sample (to over 200 °C) after room temperature storage for two months. (a) 1st cycle; (b) 2nd cycle. Heating/cooling speed: 5 °C /min; Figure S2: Heat flow versus temperature curves (DSC test). The samples were heated from room temperature to 120 °C, 140 °C, 150 °C, 165 °C and 185 °C, respectively (1st heating), and then cooled back to room temperature, followed by heating to over 240 °C (2nd heating). Heating/cooling speed: 5 °C /min; Figure S3: Shape fixity ratio (%) against programming temperature. Symbols are used for individual experimental result, and solid lines of the same color as the symbols are for the average; Figure S4: Shape fixity ratio (%) against storage time. Symbols are used for individual experimental result, and solid lines of the same color as the symbols are for the average; Figure S5: Shape recovery ratio (%) against storage time. Symbols are used for individual experimental result, and solid lines of the same color as the symbols are for the average; Figure S6: Residual strain against storage time. Symbols are used for individual experimental result, and solid lines of the same color as the symbols are for the average; Figure S7: Stress versus strain relationship in cyclic uniaxial tension (for comparison of the influence of programming strain). (I) Samples programmed at 100 °C (a) and 110 ^o^C (b); (II) samples programmed at 120 °C (a) and 130 °C (b).

## Fundings

This work is supported by the Pre-Research Foundation of Nanjing Institute of Technology, China, Natural Science Foundation of Jiangsu Province, P. R. China (BK20160773), National Natural Science Foundation of China (NSFC 21603102 and 11828201) and Outstanding Scientific and Technological Innovation Team in Colleges and Universities of Jiangsu Province, China.
