# Supplementary material for: Influence of Long-Term Storage on Shape Memory Performance and Mechanical Behavior of Pre-stretched Commercial Poly(methyl methacrylate) (PMMA)
Source: Polymers (Basel). 2019 Dec 1;11(12):1978. doi: 10.3390/polym11121978 (PMC6960707; doi:10.3390/polym11121978)
Supplement: Supplementary file 1 [file polymers-11-01978-s001.pdf]

polymers

Supplement

Influence of Long-Term Storage on Shape Memory Performance and Mechanical Behavior of Pre-stretched Commercial Poly(methyl methacrylate) (PMMA)

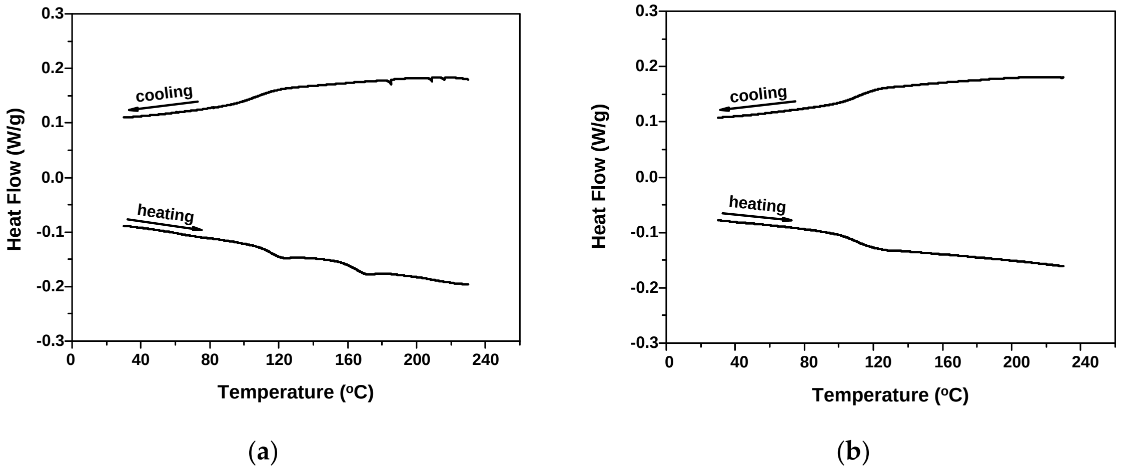

Figure S1. DSC result of pre-heated PMMA sample (to over 200 °C) after room temperature storage for two months. (**a**) 1st cycle; (**b**) 2nd cycle. Heating/cooling speed: 5 °C /min.

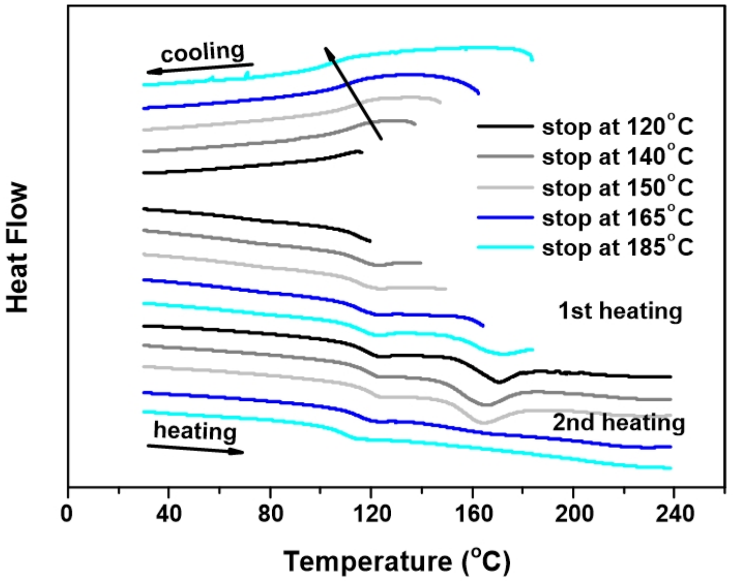

Figure S1. Heat flow versus temperature curves (DSC test). The samples were heated from room temperature to 120 °C, 140 °C, 150 °C, 165 °C and 185 °C, respectively (1st heating), and then cooled back to room temperature, followed by heating to over 240 °C (2nd heating). Heating/cooling speed: 5 °C/min.

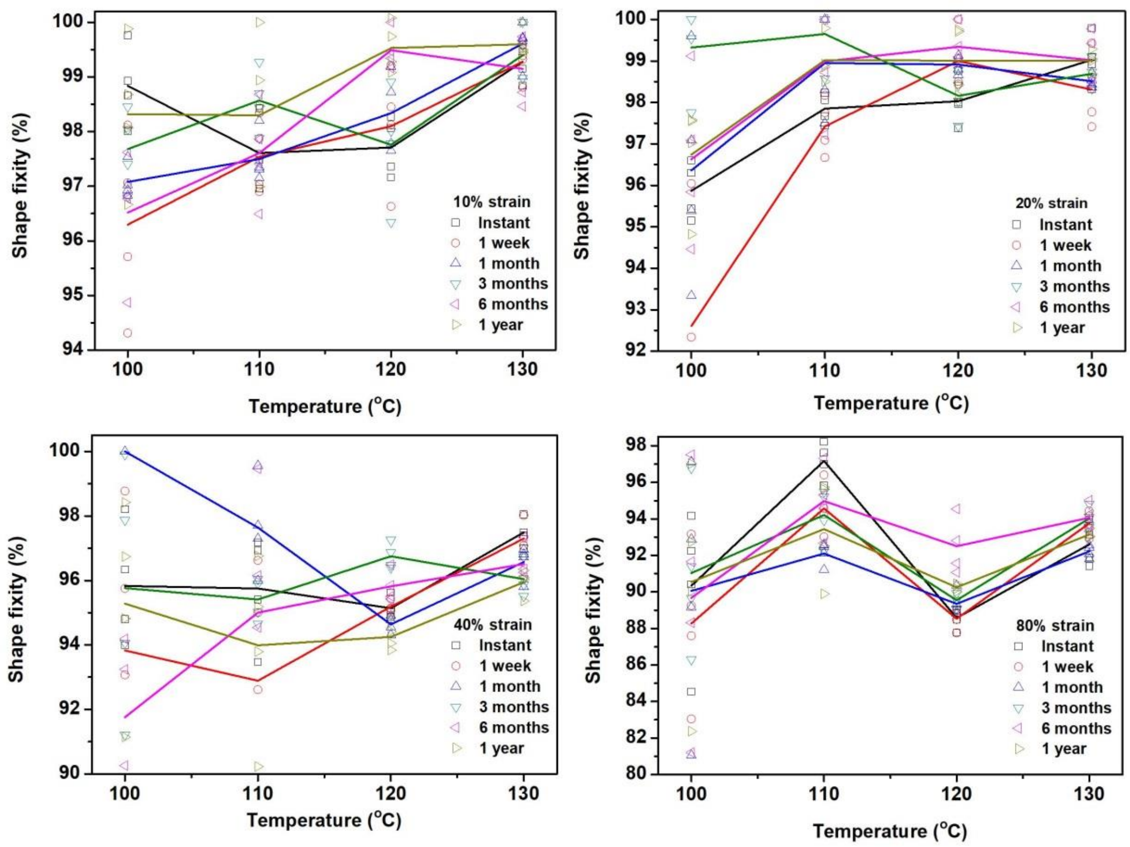

Figure S3. Shape fixity ratio (%) against programming temperature. Symbols are used for individual experimental result, and solid lines of the same color as the symbols are for the average.

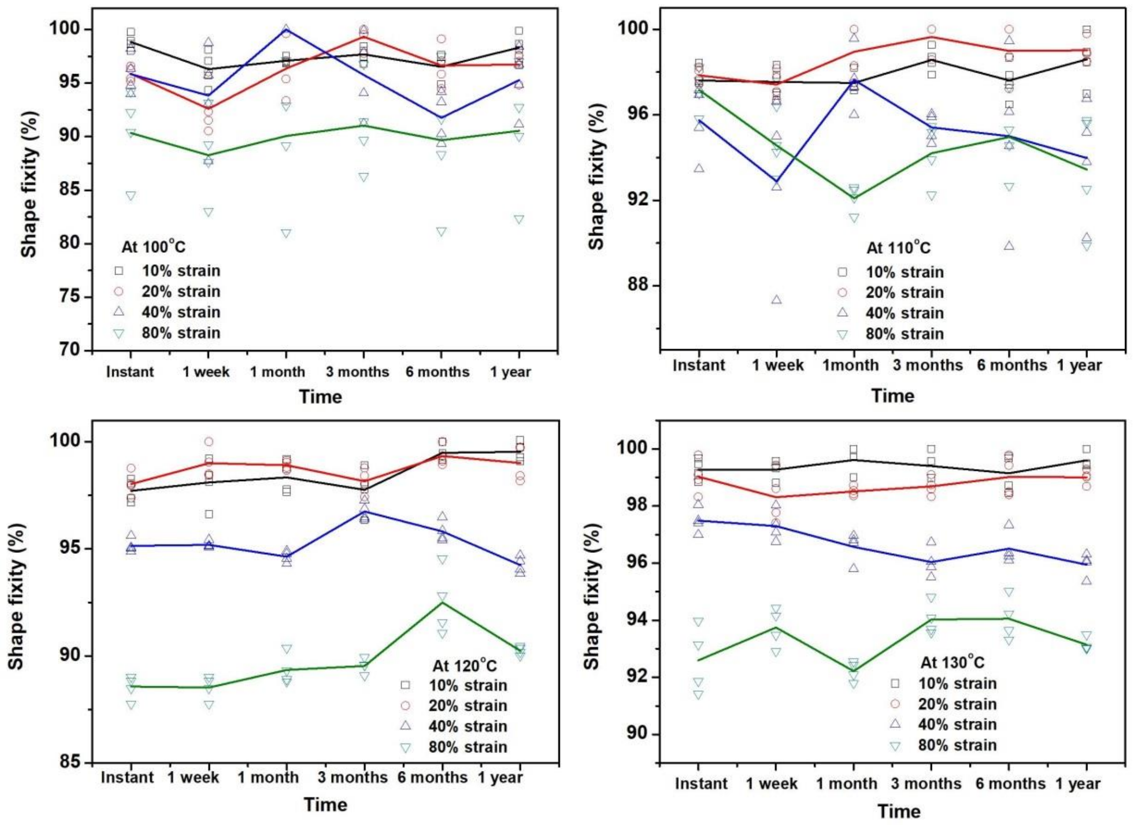

Figure S2. Shape fixity ratio (%) against storage time. Symbols are used for individual experimental result, and solid lines of the same color as the symbols are for the average.

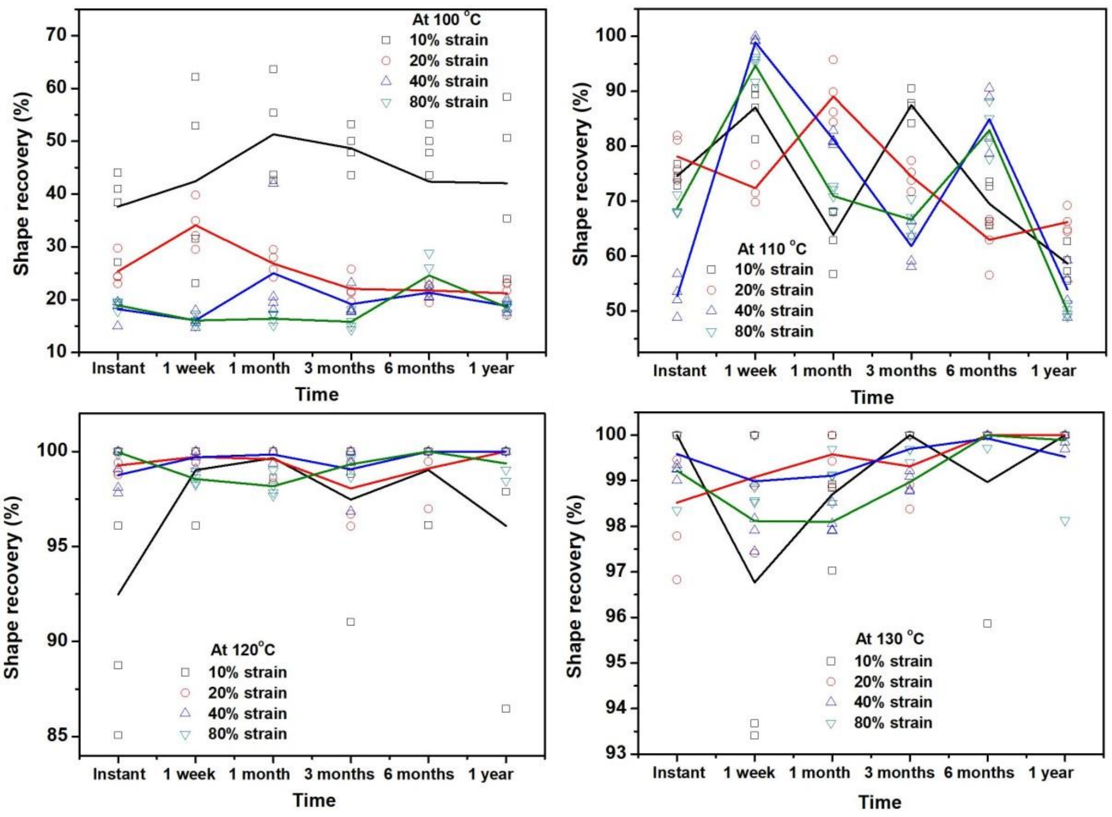

Figure S5. Shape recovery ratio (%) against storage time. Symbols are used for individual experimental result, and solid lines of the same color as the symbols are for the average.

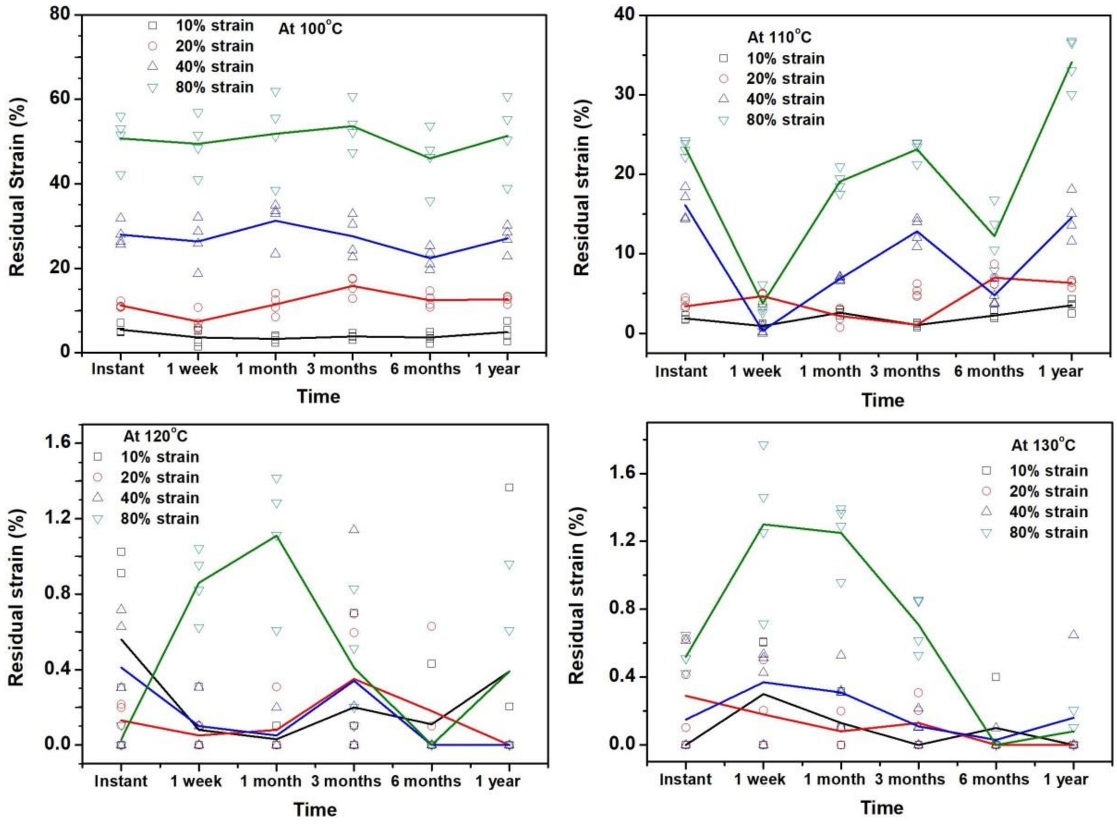

Figure S6. Residual strain against storage time. Symbols are used for individual experimental result, and solid lines of the same color as the symbols are for the average.

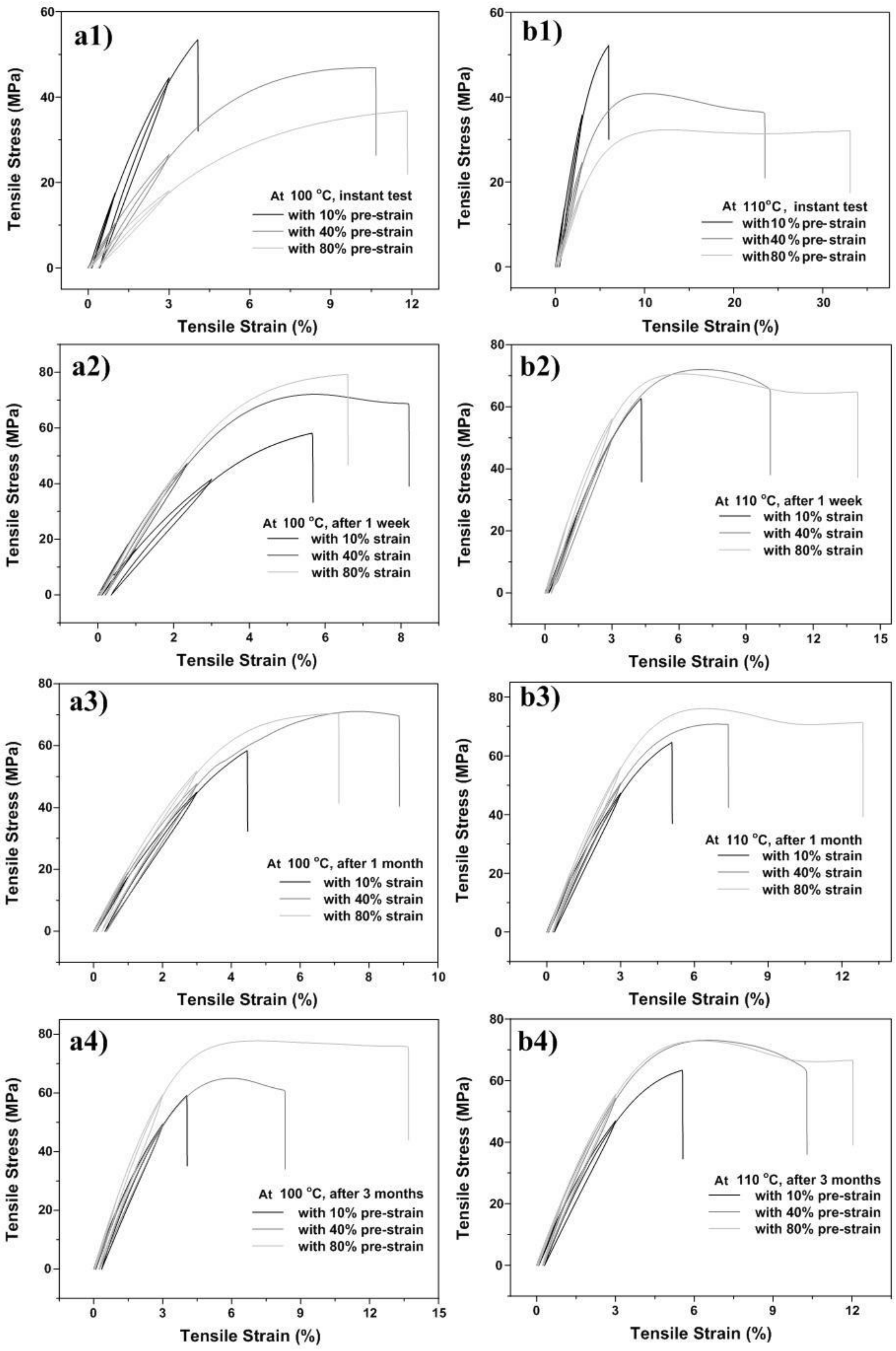
a1)
At 100 °C, instant test
with 10% pre-strain
with 40% pre-strain
with 80% pre-strain
b1)
At 110°C, instant test
with 10 % pre-strain
with 40 % pre-strain
with 80 % pre-strain
a2)
At 100 °C, after 1 week
with 10% strain
with 40% strain
with 80% strain
b2)
At 110 °C, after 1 week
with 10% strain
with 40% strain
with 80% strain
a3)
At 100 °C, after 1 month
with 10% strain
with 40% strain
with 80% strain
b3)
At 110 °C, after 1 month
with 10% strain
with 40% strain
with 80% strain
a4)
At 100 °C, after 3 months
with 10% pre-strain
with 40% pre-strain
with 80% pre-strain
b4)
At 110 °C, after 3 months
with 10% pre-strain
with 40% pre-strain
with 80% pre-strain
Tensile Stress (MPa)
Tensile Strain (%)

(I)

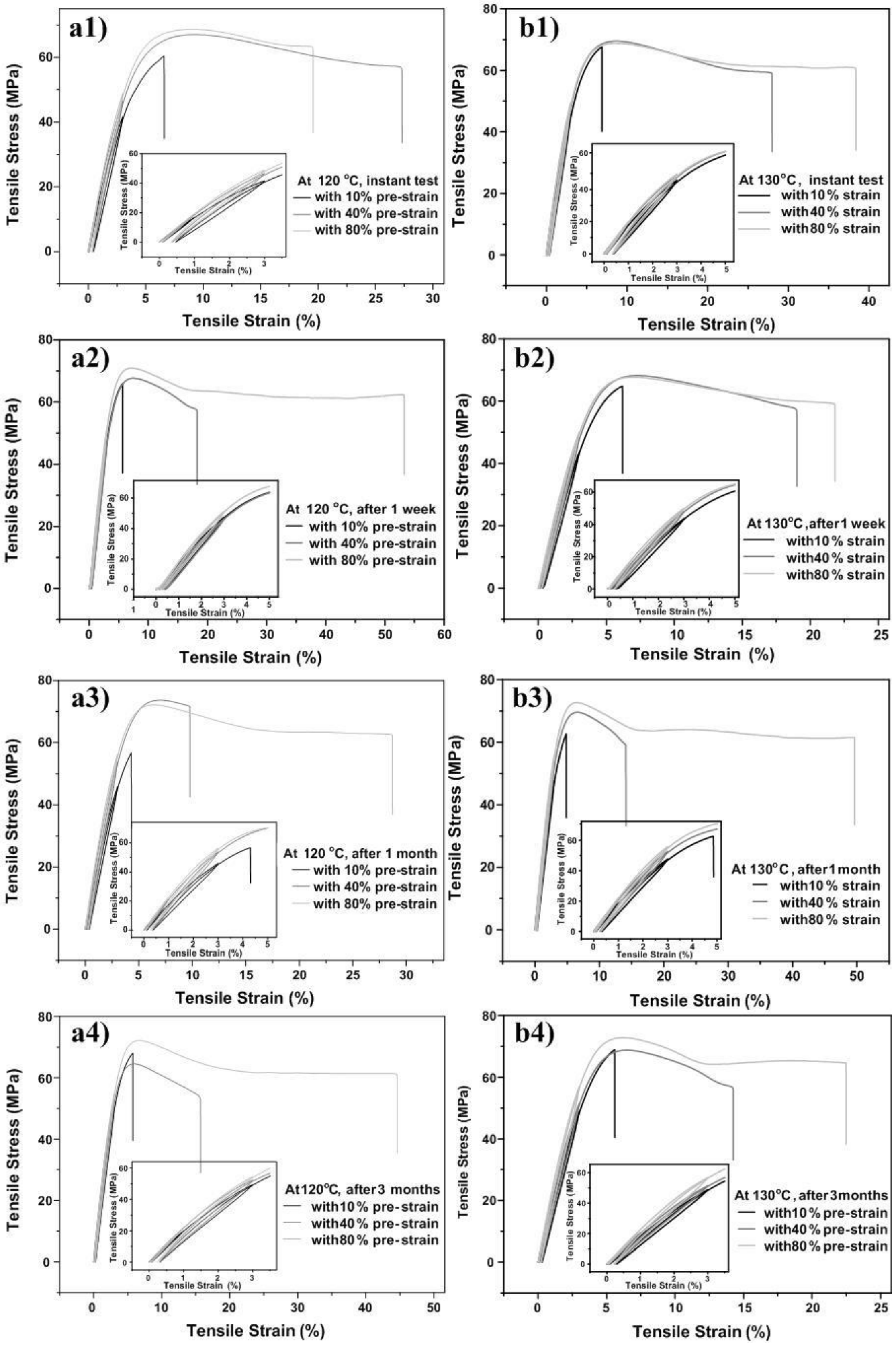

(II)

Figure S7. Stress versus strain relationship in cyclic uniaxial tension (for comparison of the influence of programming strain). (**I**) Samples programmed at 100 °C (**a**) and 110 °C (**b**); (**II**) samples programmed at 120 °C (**a**) and 130 °C (**b**).
